# 

**Published:** 1893-06-03

**Authors:** 


### SPECIAL HOSPITAL SUNDAY NUMBER.

### CONTENTS.

**A Paternal Kingdom** . . . . . . . . . . . 145

**Around the Hospitals** . . . . . . . . . . . 146

**Hospital Sweetness and Light.** By Geo. W. Potter, M.D. 147

**May the Children Help?** . . . . . . . . . 149

**How to Make Both Ends Meet.** An Interview with the Rev. Canon Palmer . . . . . . . . . . . . 150

**Annotations.**—Pains—Brains—Gains . . . . . . 151

**Notes and News.** . . . . . . . . . . 152

**A Year's Work in the Hospitals and Medical Charities of London** . . . . . . . . . . 155

**The London Hospital.**—I. By Our Special Commissioner . 153

**New Drugs and Preparations.**—Eucalyptus in the Treatment of Scarlet Fever by Inunction . . . . . . . 158

**The Institutional Workshop**—

Practical Departments.—The "Braloo" Window . . . 159

Festival Dinners and Meetings.—Hospital for Sick Children, Great Ormond Street—British Home for Incurables, Clapham 160

Construction Notes . . . . . . . . . 160

**Scraps and Gleanings** . . . . . . . . . 160

**Medical Vacancies and Appointments** . . . . . x

### EXTRA SUPPLEMENT.—THE NURSING MIRROR.

**News from the Nursing World.**—The Royal Wedding—The Nurse Dolls—The late Dr. Hadden—More Work for Competent Women—Freedom and Fresh Air—Ministering Children at Lancaster—Pennies Well Applied—An Enthusiastic Meeting—Contemplated Changes at Leicester—Fleeting Glories—Short Items—"The Hospital" Endowed Bed . xci

**Sanitation for Nurses.** By A Sanitary Inspector. VIII.—Vaccination in Various Countries . . . . . . xciii

**Hospital Nursing in the North of India.**—By Our Indian Correspondent.—A Visit to a Cantonment Hospital . xciv

**Fair and Unfair Expenses** . . . . . . . xciv

**Registration of Nurses.**—The Nature of the Opposition . xcv

**Prince Alfred Hospital, Sydney.**—The New Nurses' Home xcv

**For Reading to the Sick.**—Music . . . . . . xcvi

**Appointments** . . . . . . . . . . xcvi

**Wants and Workers** . . . . . . . . . xcvi

**Examinations for Probationers.**—By A Victim . . xcvii

**Notes and Queries** . . . . . . . . . xcvii

**Sent** . . . . . . . . . . . . xcviii

**The Book World for Women and Nurses** . . . . xcix

